# Supplementary material for: Dihydroceramide- and ceramide-profiling provides insights into human cardiometabolic disease etiology
Source: Nat Commun. 2022 Feb 17;13:936. doi: 10.1038/s41467-022-28496-1 (PMC8854598; doi:10.1038/s41467-022-28496-1)
Supplement: Supplementary file 1 — Supplementary Information [file 41467_2022_28496_MOESM1_ESM.pdf]

SUPPLEMENTARY INFORMATION

Dihydroceramide- and ceramide-profiling provides insights into human cardiometabolic disease etiology

Wittenbecher C, Cuadrat R, Johnston L, Eichelmann F, Jäger S, Kuxhaus O, Prada M, Del Greco F, Hicks AA, Hoffman P, Krumsiek J, Hu FB, Schulze MB

Contents

Supplementary Tables ... 3

Supplementary Table 1: Lipid nomenclature. Matching short name used in this study to common name and lipid maps identifier ... 3

Supplementary Table 2: Baseline characteristics across subgroups according to sex-specific total plasma ceramide concentration ... 4

Supplementary Table 3: Baseline characteristics across subgroups according to sex-specific total plasma dihydroceramide concentration ... 6

Supplementary Table 4: Baseline characteristics in participants without and with incident T2D ... 8

Supplementary Table 5: Baseline characteristics in participants without and with incident CVD ... 9

Supplementary Table 6: Minimally adjusted & confounder-adjusted T2D- and CVD-hazard ratios for single (dh)ceramides ... 10

Supplementary Table 7: *Network-adjusted T2D hazard ratios (HR)*. Range of HRs additionally adjusted for all subsets of direct neighbors in the (dh)ceramides-network, according to the *NetCoupler-algorithm.* ... 12

Supplementary Table 8: *Network-adjusted CVD hazard ratios (HR)*. Range of HRs additionally adjusted for all subsets of direct neighbors in the (dh)ceramides-network, according to the *NetCoupler-algorithm.* ... 13

Supplementary Table 9: Joint association of the selected (dh)ceramides with direct effects on CVD and T2D, additionally adjusted for HDL-cholesterol. ... 14

Supplementary Table 10: Joint association of the selected (dh)ceramides with direct effects on CVD and T2D, excluding participants on lipid-lowering medication at baseline. ... 15

Supplementary Table 11: Joint association of the selected (dh)ceramides with direct effects on CVD and T2D, excluding participants with CVD or T2D incidence within the first two years of follow-up ... 16

Supplementary Table 12: Matching pathway-names in Supplementary Figure 3 to the original database ... 17

Supplementary Figures ... 20

Supplementary Figure 1: Biological reliability of repeated ceramide and dihydroceramide measurements. ... 20

Supplementary Figure 2: Correlations (lower left triangle) and partial correlations (upper left triangle) among (dh)ceramides. ... 21

Supplementary Figure 3: Pathway enrichment analysis. ... 22

Additional description of Supplementary Figure 3. ... 22

Supplementary Figure 4: The NetCoupler algorithm ... 23

Supplementary Tables

Supplementary Table 1: Lipid nomenclature. Matching short name used in this study to common name and lipid maps identifier

Short name	Common name	LIPID MAPS ID
dhCer14:0	Dihydroceramide (d18:0/14:0)	LMSP02020016
dhCer16:0	Dihydroceramide (d18:0/16:0)	LMSP02020001
dhCer18:0	Dihydroceramide (d18:0/18:0)	LMSP02020008
dhCer18:1	Dihydroceramide (d18:0/18:1)	LMSP02020015
dhCer20:0	Dihydroceramide (d18:0/20:0)	LMSP02020009
dhCer20:1	Dihydroceramide (d18:0/20:1)	NA
dhCer22:0	Dihydroceramide (d18:0/22:0)	LMSP02020010
dhCer22:1	Dihydroceramide (d18:0/22:1)	NA
dhCer22:2	Dihydroceramide (d18:0/22:2)	NA
dhCer24:0	Dihydroceramide (d18:0/24:0)	LMSP02020012
dhCer24:1	Dihydroceramide (d18:0/24:1)	LMSP02020011
dhCer26:0	Dihydroceramide (d18:0/26:0)	LMSP02020014
dhCer26:1	Dihydroceramide (d18:0/26:1)	LMSP02020013
Cer14:0	Ceramide (d18:1/14:0)	LMSP02010001
Cer16:0	Ceramide (d18:1/16:0)	LMSP02010004
Cer18:0	Ceramide (d18:1/18:0)	LMSP02010006
Cer18:1	Ceramide (d18:1/18:1)	LMSP02010003
Cer20:0	Ceramide (d18:1/20:0)	LMSP02010007
Cer20:1	Ceramide (d18:1/20:1)	NA
Cer22:0	Ceramide (d18:1/22:0)	LMSP02010008
Cer22:1	Ceramide (d18:1/22:1)	NA
Cer24:0	Ceramide (d18:1/24:0)	LMSP02010012
Cer24:1	Ceramide (d18:1/24:1)	LMSP02010009
Cer26:0	Ceramide (d18:1/26:0)	LMSP02010011
Cer26:1	Ceramide (d18:1/26:1)	LMSP02010010
(dh)ceramides	Ceramides and Dihydroceramides	

NA: not available. LIPID MAPS IDs from http://www.lipidmaps.org/ (downloaded 2019-12-19).

Supplementary Table 2: Baseline characteristics across subgroups according to sex-specific total plasma ceramide concentration

	Subgroups according to quintiles of total ceramide distribution				
	**<q1**	**q1-q2**	**q2-q3**	**q3-q4**	**>q4**
N	228	227	227	227	228
Total ceramides men [nM]	35.1-80.2	80.4-91.6	91.8-104.8	105-118.4	118.5-193.6
Total ceramides women [nM]	33.6-69.5	69.8-80	80-92.7	92.8-107.9	108.2-208.6
Women	61%	61%	60%	61%	61%
Age [years]	44 (39.8, 51.5)	46.7 (41.4, 56.3)	51.3 (43.7, 58.3)	52.9 (43, 58.9)	54.3 (45.9, 59.4)
Height	169 (163, 175.3)	167.4 (162.4, 173.5)	167.2 (161.2, 174.4)	166.2 (160.8, 172.5)	164.5 (159.2, 173.2)
Waist circumference	80 (71, 89.6)	82 (72.5, 93)	86.5 (75.5, 94)	86 (77.2, 94)	89 (80, 96.1)
Leisure-time physical activity (h/week)	4.5 (2.4, 7.6)	5 (2.8, 8)	5 (2, 8.5)	5.5 (2, 8.2)	4.5 (2, 8.1)
Energy intake (MJ/day)	8196 (6897, 10385)	8657 (6776, 10433)	8229 (6595, 10325)	8301.5 (6865, 10245)	8525 (6910, 10085)
Fasted (>8h)	17%	15%	15%	13%	15%
Education					
vocational training or lower	21%	27%	25%	22%	25%
technical college	44%	43%	37%	33%	32%
university	36%	30%	39%	46%	43%
Smoking					
never	47%	48%	46%	49%	53%
former	36%	32%	35%	26%	28%
current, <= 20 U/day	13%	16%	14%	19%	12%
current, >20 U/day	4%	4%	5%	6%	7%
Alcohol consumption					
none	40%	33%	41%	43%	39%
low	15%	19%	17%	17%	16%
moderately low	20%	20%	19%	19%	19%
moderately high	20%	24%	18%	15%	20%
high	3%	2%	3%	4%	2%
very high	2%	1%	1%	2%	4%
Blood pressure					

systolic	122.2 (113, 132.6)	126.5 (116.8, 140.2)	126 (117.5, 139.2)	129.5 (119, 140.8)	133 (120.5, 143)
diastolic	80 (74, 87)	82 (75.8, 90.5)	83 (76, 90.5)	83.5 (76.5, 90.8)	85.5 (78, 92)
Blood triglycerides	79.1 (61, 107.7)	91.6 (66.3, 125.6)	104.5 (77.3, 149.5)	120.9 (83.1, 184.8)	160.2 (109, 227)
Blood cholesterol	176.1 (155.3, 197.6)	196 (173.7, 215.1)	200 (182.2, 223.2)	216.2 (191.8, 239.5)	238.9 (213.6, 263)
Medication					
ASS	9%	7%	11%	11%	11%
antihypertensive drugs	11%	20%	24%	22%	22%
lipid lowering drugs	4%	4%	6%	7%	5%

Distribution of potential confounders at recruitment (baseline) in the random subcohort, which is representative for the full EPIC-Potsdam cohort

Supplementary Table 3: Baseline characteristics across subgroups according to sex-specific total plasma dihydroceramide concentration

	Subgroups according to quintiles of the total dh-ceramide distribution				
	<q1	q1-q2	q2-q3	q3-q4	>q4
N	228	227	227	227	228
Total dh-ceramides men [nM]	28.3-42.4	42.5-46.1	46.3-49.8	49.9-57.6	57.6-97.3
Total dh-ceramides women [nM]	28.8-38.9	39-42.9	43-46.9	46.9-53.2	53.3-129.7
Women	60%	61%	60%	61%	61%
Age [years]	45.1 (40.1, 55.5)	47.9 (41.9, 57)	50.8 (42.1, 58.2)	52.2 (44.1, 57.7)	53.2 (44, 59.1)
Height	167.5 (161.7, 174.5)	167.8 (162.1, 174.1)	167 (161.8, 173.9)	166.6 (161.2, 173.3)	164.5 (159.6, 172.5)
Waist circumference	80 (71.2, 90)	82.8 (73, 91.5)	86 (76.8, 94)	87.5 (77.2, 94)	88 (78.5, 96.6)
Leisure-time PA (h/week)	4.5 (2.2, 7.5)	5 (2.5, 8.4)	5 (2.5, 8.5)	5 (2, 8.5)	4 (1.5, 8)
Energy intake (MJ/day)	8159 (6857, 10450)	8545 (6792, 10364)	8317 (6654, 10496)	8136 (6761, 10162)	8788 (6905, 10072)
Fasted (>8h)	18%	17%	17%	11%	11%
Education					
vocational training or lower	21%	28%	23%	23%	24%
technical college	43%	41%	41%	33%	30%
university	36%	31%	37%	44%	46%
Smoking					
never	48%	46%	50%	51%	47%
former	34%	28%	31%	35%	29%
current, <= 20 U/day	12%	19%	17%	10%	16%
current, >20 U/day	5%	7%	2%	5%	7%
Alcohol consumption					
none	3%	3%	2%	3%	4%
low	44%	39%	38%	40%	36%
moderately low	14%	16%	18%	17%	19%
moderately high	22%	16%	21%	19%	19%
high	18%	23%	19%	18%	19%
very high	0%	2%	1%	3%	4%
Blood pressure					

systolic	122.5 (112.8, 134)	128.2 (117, 138.2)	125.5 (118.5, 138)	131 (117, 141)	131 (119.5, 144)
diastolic	80 (73.8, 87.2)	83.5 (76, 90.5)	82.5 (76, 90.5)	84.5 (77, 92.2)	84.5 (77.5, 91.5)
Blood triglycerides	78.7 (61.6, 106.8)	97.7 (69.8, 127.6)	108.1 (79.2, 159.3)	116.2 (80.3, 180.7)	158.9 (107, 236.3)
Blood cholesterol	181.4 (158.7, 201.5)	199.8 (179, 219.3)	205.4 (183.7, 227.3)	215.1 (182.2, 236.7)	230.7 (193.8, 260.4)
Medication					
ASS	8%	8%	12%	11%	11%
antihypertensive drugs	14%	18%	21%	22%	25%
lipid lowering drugs	4%	6%	4%	6%	7%

Distribution of potential confounders at recruitment (baseline) in the random subcohort, which is representative for the full EPIC-Potsdam cohort

Supplementary Table 4: Baseline characteristics in participants without and with incident T2D

Covariables	Non-cases	Incident T2D cases
N	1111	775
Women	61%	42%
Age [years]	49.3 (41.9, 57.5)	56.5 (49.5, 60.9)
Height	167.1 (161.1, 173.8)	168.7 (162.5, 175)
Waist circumference	84.5 (75, 93)	100 (92, 107.5)
Leisure-time PA (h/week)	5 (2, 8)	4.5 (1.5, 8.5)
Energy intake (MJ/day)	8454 (6804, 10287)	8845 (7170, 10710)
Fasted (>8h)	15%	14%
Education		
vocational training or lower	38%	46%
technical college	24%	23%
university	38%	31%
Smoking		
never	49%	34%
former	31%	44%
current, <= 20 U/day	15%	12%
current, >20 U/day	5%	9%
Alcohol consumption		
none	3%	4%
low	39%	37%
moderately low	19%	20%
moderately high	20%	18%
high	17%	17%
very high	2%	4%
Blood pressure		
systolic	82.5 (76, 90.3)	89.5 (82.5, 95.5)
diastolic	127 (116.5, 139.5)	138 (127.5, 151)
Blood triglycerides	203.4 (177.9, 230.1)	212.5 (186.6, 239.5)
Blood cholesterol	105.6 (74.4, 161.2)	170.2 (127.9, 239.7)
Medication		
ASS	10%	13%
Antihypertensive drugs	19%	39%
Lipid lowering drugs	5%	10%

Distribution of potential confounders at recruitment (baseline) in participants without and with incident T2D during follow-up.

Supplementary Table 5: Baseline characteristics in participants without and with incident CVD

Covariables	Non-cases	Incident CVD cases
N	1120	551
Women	63%	34%
Age [years]	49.3 (41.9, 57.5)	57.9 (52.3, 62.2)
Height	166.8 (161, 173.5)	169.4 (163.7, 175)
Waist circumference	84.5 (75, 93.5)	93 (85, 101)
Leisure-time PA (h/week)	4.5 (2, 8)	5 (2, 10)
Energy intake (MJ/day)	8411.9 (6794.5, 10298.9)	8893.9 (7277.1, 10514)
Fasted (>8h)	15%	18%
Education		
vocational training or lower	38%	40%
technical college	24%	25%
university	38%	35%
Smoking		
never	49%	32%
former	31%	33%
current, <= 20 U/day	15%	21%
current, >20 U/day	5%	14%
Alcohol consumption		
none	3%	6%
low	40%	34%
moderately low	19%	18%
moderately high	19%	18%
high	16%	20%
very high	2%	4%
Blood pressure		
systolic	82.5 (76, 90)	87.5 (81.5, 95.5)
diastolic	127 (116.5, 139.5)	137.5 (126.5, 152.5)
Blood triglycerides	203.4 (177.7, 229)	216 (189.5, 243.6)
Blood cholesterol	105.7 (74.4, 161)	140.9 (94.6, 209.1)
Medication		
ASS	18%	34%
Antihypertensive drugs	9%	9%
Lipid lowering drugs	4%	7%

Distribution of potential confounders at recruitment (baseline) in participants without and with incident CVD during follow-up.

Supplementary Table 6: Minimally adjusted & confounder-adjusted T2D- and CVD-hazard ratios for single (dh)ceramides

	Model 1		Model 2		Model 3	
(dh)Cer	HR (95%CI)	FDR	HR (95%CI)	FDR	HR (95%CI)	FDR
**CVD**						
Cer14:0	1.47 (1.29, 1.67)	1.6E-08	1.44 (1.22, 1.69)	5.5E-05	1.24 (1.04, 1.48)	0.197
Cer16:0	1.55 (1.35, 1.77)	9.0E-10	1.48 (1.22, 1.8)	2.4E-04	1.36 (1.04, 1.79)	0.211
Cer18:0	1.57 (1.38, 1.78)	9.8E-11	1.37 (1.16, 1.62)	5.2E-04	1.20 (0.97, 1.50)	0.412
Cer18:1	1.49 (1.31, 1.7)	6.9E-09	1.3 (1.11, 1.52)	0.002	1.11 (0.94, 1.32)	0.461
Cer20:0	1.49 (1.31, 1.69)	5.7E-09	1.3 (1.1, 1.54)	0.004	1.06 (0.79, 1.41)	0.907
Cer20:1	1.15 (1.02, 1.31)	0.027	1.11 (0.96, 1.27)	0.163	1.01 (0.88, 1.16)	0.959
Cer22:0	1.5 (1.32, 1.72)	6.9E-09	1.36 (1.14, 1.63)	0.002	1.26 (0.77, 2.05)	0.554
Cer22:1	1.23 (1.1, 1.38)	5.4E-04	1.05 (0.92, 1.2)	0.473	0.89 (0.76, 1.05)	0.461
Cer24:0	1.43 (1.25, 1.63)	3.7E-07	1.36 (1.14, 1.62)	0.001	1.02 (0.63, 1.65)	0.959
Cer24:1	1.46 (1.28, 1.67)	4.6E-08	1.25 (1.05, 1.48)	0.015	0.82 (0.60, 1.11)	0.461
Cer26:0	1.19 (1.07, 1.34)	0.002	1.21 (1.07, 1.38)	0.005	1.00 (0.86, 1.17)	0.959
Cer26:1	1.23 (1.08, 1.4)	0.002	1.08 (0.92, 1.26)	0.373	0.86 (0.72, 1.04)	0.412
dhCer14:0	1.31 (1.15, 1.48)	4.8E-05	1.22 (1.05, 1.41)	0.015	1.01 (0.85, 1.20)	0.959
dhCer16:0	1.42 (1.27, 1.57)	8.2E-10	1.34 (1.18, 1.52)	5.5E-05	1.07 (0.88, 1.29)	0.756
dhCer18:0	1.29 (1.17, 1.43)	3.7E-07	1.27 (1.13, 1.41)	1.3E-04	1.10 (0.93, 1.30)	0.475
dhCer18:1	1.46 (1.29, 1.65)	6.9E-09	1.31 (1.13, 1.51)	8.6E-04	1.12 (0.94, 1.33)	0.461
dhCer20:0	1.47 (1.31, 1.65)	8.8E-10	1.4 (1.21, 1.62)	5.5E-05	1.19 (0.96, 1.48)	0.412
dhCer20:1	1.43 (1.27, 1.6)	6.9E-09	1.31 (1.13, 1.52)	8.6E-04	1.11 (0.92, 1.35)	0.490
dhCer22:0	1.55 (1.37, 1.75)	9.8E-11	1.41 (1.18, 1.69)	5.2E-04	1.03 (0.77, 1.37)	0.959
dhCer22:1	1.37 (1.20, 1.56)	2.1E-06	1.21 (1.04, 1.41)	0.015	0.97 (0.81, 1.15)	0.907
dhCer22:2	1.52 (1.33, 1.74)	2.5E-09	1.62 (1.36, 1.93)	1.2E-06	1.44 (1.15, 1.8)	0.031
dhCer24:0	1.40 (1.23, 1.6)	8.1E-07	1.29 (1.09, 1.54)	0.005	0.75 (0.53, 1.07)	0.412
dhCer24:1	1.46 (1.29, 1.66)	1.4E-08	1.31 (1.12, 1.53)	0.001	0.93 (0.74, 1.17)	0.766
dhCer26:0	1.07 (0.96, 1.2)	0.225	1.10 (0.97, 1.24)	0.158	0.93 (0.81, 1.08)	0.554
dhCer26:1	1.22 (1.07, 1.39)	0.003	1.15 (1.00, 1.32)	0.051	0.89 (0.75, 1.07)	0.461
**T2D**						
Cer14:0	1.09 (0.99, 1.21)	0.095	1.08 (0.95, 1.24)	0.329	1.03 (0.88, 1.21)	0.862
Cer16:0	1.27 (1.14, 1.42)	2.9E-05	1.03 (0.88, 1.19)	0.791	1.03 (0.81, 1.31)	0.881
Cer18:0	1.86 (1.65, 2.08)	1.5E-24	1.28 (1.09, 1.49)	0.011	1.45 (1.17, 1.8)	0.006
Cer18:1	1.32 (1.19, 1.46)	1.5E-07	1.13 (0.99, 1.29)	0.143	1.10 (0.95, 1.27)	0.438
Cer20:0	1.55 (1.39, 1.73)	1.1E-14	1.13 (0.98, 1.31)	0.192	1.35 (1.03, 1.76)	0.127
Cer20:1	1.13 (1.03, 1.25)	0.015	1.06 (0.94, 1.19)	0.430	1.02 (0.9, 1.15)	0.881
Cer22:0	1.52 (1.36, 1.7)	3.5E-13	1.13 (0.97, 1.31)	0.192	2.25 (1.44, 3.53)	0.005
Cer22:1	1.26 (1.14, 1.4)	1.9E-05	1.12 (0.98, 1.26)	0.178	1.14 (0.98, 1.31)	0.246
Cer24:0	1.19 (1.07, 1.33)	0.002	0.94 (0.81, 1.08)	0.431	0.56 (0.37, 0.85)	0.030
Cer24:1	1.46 (1.30, 1.63)	1.30E-10	1.03 (0.89, 1.19)	0.791	1.06 (0.8, 1.41)	0.862
Cer26:0	1.09 (1.00, 1.18)	0.074	1.09 (0.98, 1.2)	0.199	1.01 (0.9, 1.14)	0.881
Cer26:1	1.09 (0.98, 1.22)	0.135	0.89 (0.77, 1.04)	0.204	0.83 (0.70, 0.99)	0.154
dhCer14:0	1.23 (1.11, 1.37)	8.1E-05	1.1 (0.97, 1.25)	0.204	1.01 (0.87, 1.17)	0.933
dhCer16:0	1.28 (1.17, 1.40)	1.5E-07	1.13 (0.99, 1.28)	0.143	0.98 (0.83, 1.16)	0.881
dhCer18:0	1.28 (1.17, 1.40)	4.4E-08	1.19 (1.07, 1.32)	0.011	1.09 (0.95, 1.25)	0.440
dhCer18:1	1.43 (1.30, 1.58)	3.6E-12	1.17 (1.03, 1.33)	0.055	1.09 (0.94, 1.26)	0.476

dhCer20:0	1.53 (1.39, 1.69)	2.6E-16	1.34 (1.18, 1.52)	8.6E-05	1.42 (1.17, 1.72)	0.005
dhCer20:1	1.49 (1.35, 1.65)	1.5E-14	1.22 (1.07, 1.39)	0.011	1.12 (0.95, 1.32)	0.422
dhCer22:0	1.79 (1.61, 2.00)	3.0E-24	1.30 (1.12, 1.51)	0.005	1.58 (1.21, 2.07)	0.006
dhCer22:1	1.45 (1.31, 1.61)	2.7E-12	1.17 (1.03, 1.33)	0.055	1.08 (0.93, 1.26)	0.512
dhCer22:2	1.74 (1.55, 1.96)	8.4E-20	1.25 (1.07, 1.46)	0.020	1.19 (0.99, 1.45)	0.218
dhCer24:0	1.35 (1.21, 1.5)	1.5E-07	1.02 (0.89, 1.17)	0.819	0.90 (0.67, 1.19)	0.671
dhCer24:1	1.56 (1.40, 1.73)	3.3E-15	1.17 (1.02, 1.34)	0.077	1.10 (0.90, 1.35)	0.570
dhCer26:0	1.06 (0.97, 1.15)	0.200	1.08 (0.97, 1.2)	0.214	0.97 (0.86, 1.10)	0.862
dhCer26:1	1.01 (0.91, 1.11)	0.918	1.01 (0.89, 1.14)	0.879	0.89 (0.77, 1.03)	0.281

Hazard Ratios (HR) for cardiovascular diseases (CVD) and type 2 diabetes (T2D).

Model 1: adjusted for age (strata variable) and sex.

Model 2: adjusted age (strata variable), sex, height, waist circumference, leisure-time physical activity, fasting status, antihypertensive medication, lipid-lowering medication, aspirin, total energy intake, smoking, alcohol consumption, educational attainment, plasma concentrations of triglycerides, total cholesterol, and systolic and diastolic blood pressure; baseline-prevalent T2D cases were excluded from the diabetes risk model, and adjusted for in the CVD risk model.

Model 3: additionally adjusted for total ceramide and total dihydro-ceramide concentrations.

FDR: false discovery rate-controlled p-values (likelihood ratio test, two-sided), adjusted for the number of available ceramides and dihydroceramides.

Supplementary Table 7: ***Network-adjusted T2D hazard ratios (HR)*****. Range of HRs additionally adjusted for all subsets of direct neighbors in the (dh)ceramides-network, according to the** ***NetCoupler-algorithm*****.**

	Confounder- & Network-adjusted Median HR	Min HR	Max HR	Max P	Round
**Cer18:0**	**1.64**	**1.51**	**1.8**	**6.34E-05**	**1**
**dhCer20:0**	**1.505**	**1.34**	**1.6**	**2.58E-04**	**1**
**Cer22:0**	**2.045**	**1.66**	**2.69**	**0.00355**	**2**
**dhCer26:1**	**0.805**	**0.79**	**0.83**	**0.00878**	**2**
**Cer20:0**	**0.58**	**0.58**	**0.61**	**0.0154**	**3**
**dhCer22:2**	**1.205**	**1.18**	**1.23**	**0.0481**	**3**
Cer18:1	0.86	0.85	0.87	0.0631	
Cer16:0	0.8	0.78	0.82	0.0782	
dhCer14:0	0.87	0.86	0.88	0.104	
dhCer18:0	0.905	0.87	0.93	0.148	
dhCer16:0	0.935	0.88	1.07	0.197	
Cer26:0	1.085	1.07	1.09	0.224	
Cer20:1	0.92	0.87	0.98	0.228	
dhCer24:1	1.125	1.06	1.16	0.241	
dhCer22:0	1.17	1.15	1.2	0.268	
dhCer20:1	0.965	0.9	1.02	0.291	
Cer24:1	1.145	1.1	1.19	0.32	
Cer24:0	0.69	0.61	0.77	0.33	
dhCer26:0	1.035	1.01	1.06	0.37	
Cer14:0	0.995	0.93	1.04	0.402	
dhCer18:1	1.01	0.94	1.05	0.453	
Cer26:1	1.035	1.02	1.05	0.613	
dhCer24:0	1.105	0.97	1.25	0.828	
dhCer22:1	1.03	0.99	1.07	0.867	
Cer22:1	1.04	1.01	1.07	0.896	

Hazard ratios (HR) for T2D. Median, Minimum (Min HR) and Maximum (Max HR) of all HRs, and highest p-value (Max P; likelihood ratio test, two-sided) across models adjusted for all possible combinations of direct network-neighbors, Median HR). Models were always adjusted for the *a priori*-defined confounder set and the direct effects identified in the previous round. Round: number of iterations of the adjustment for direct neighbors, always additionally adjusting for direct effects identified in the previous round.

The *a priori*-defined confounder set: age (strata variable), sex, height, waist circumference, leisure-time physical activity, fasting status, antihypertensive medication, lipid-lowering medication, aspirin, total energy intake, smoking, alcohol consumption, educational attainment, plasma concentrations of triglycerides, total cholesterol, total ceramide- and dihydro-ceramide-concentrations, and systolic and diastolic blood pressure.

Supplementary Table 8: ***Network-adjusted CVD hazard ratios (HR)*****.** Range of HRs additionally adjusted for all subsets of direct neighbors in the (dh)ceramides-network, according to the *NetCoupler-algorithm*.

(dh)ceramide	Confounder- & Network-adjusted Median HR	Min HR	Max HR	Max P	Round
dhCer22:2	1.435	1.4	1.47	0.00184	1
Cer16:0	1.43	1.33	1.54	0.00535	1
Cer26:1	0.85	0.85	0.85	0.08	
Cer22:0	1.71	1.44	2.02	0.145	
dhCer26:1	0.87	0.83	0.91	0.146	
dhCer24:0	0.71	0.65	0.78	0.167	
dhCer22:1	0.85	0.8	0.92	0.167	
dhCer14:0	0.81	0.77	0.89	0.212	
dhCer20:0	1.26	1.15	1.36	0.229	
Cer18:0	1.205	1.14	1.27	0.253	
Cer14:0	1.175	1.1	1.26	0.312	
Cer26:0	1.11	1.09	1.13	0.337	
dhCer24:1	0.97	0.89	1.04	0.346	
Cer24:1	0.945	0.84	1.02	0.352	
dhCer18:0	1.11	1.08	1.13	0.405	
Cer22:1	0.89	0.85	0.94	0.436	
Cer20:0	0.92	0.71	1.09	0.754	
dhCer26:0	0.98	0.94	1.02	0.808	
dhCer20:1	1.025	0.94	1.12	0.828	
dhCer16:0	0.94	0.9	0.98	0.89	
dhCer22:0	0.955	0.83	1.05	0.902	
Cer18:1	1	0.97	1.02	0.911	
dhCer18:1	1.035	0.99	1.1	0.947	
Cer24:0	1.315	1.01	1.64	0.961	
Cer20:1	1.03	0.99	1.06	0.982	

Hazard ratios (HR) for CVD. Median, Minimum (Min HR) and Maximum (Max HR) of all HRs, and highest p-value (Max P; likelihood ratio test, two-sided) across models adjusted for all possible combinations of direct network-neighbors, Median HR). Models were always adjusted for the *a priori*-defined confounder set and the direct effects identified in the previous round. Round: number of iterations of the adjustment for direct neighbors, always additionally adjusting for direct effects identified in the previous round.

The *a priori*-defined confounder set: age (strata variable), sex, height, waist circumference, leisure-time physical activity, fasting status, antihypertensive medication, lipid-lowering medication, aspirin, total energy intake, smoking, alcohol consumption, educational attainment, plasma concentrations of triglycerides, total cholesterol, total ceramide- and dihydro-ceramide-concentrations, systolic and diastolic blood pressure, and baseline-prevalent T2D cases.

Supplementary Table 9: Joint association of the selected (dh)ceramides with direct effects on CVD and T2D, additionally adjusted for HDL-cholesterol.

(dh)ceramide	HR (95%CI)	p-value
**CVD**		
dhCer22:2	1.66 (1.32, 2.08)	1.44E-05
Cer16:0	1.54 (1.16, 2.04)	0.0028
**T2D**		
Cer18:0	1.77 (1.27, 2.45)	6.85E-04
dhCer20:0	1.27 (1.03, 1.56)	0.0241
Cer22:0	2.1 (1.27, 3.46)	0.0037
dhCer26:1	0.86 (0.74, 1)	0.0515
Cer20:0	0.66 (0.43, 1.02)	0.0634
dhCer22:2	1.32 (1.07, 1.62)	0.0090

Hazard ratio (HR) per one standard deviation higher plasma concentration from the EPIC-Potsdam cohort.

Risk estimates are from a Cox proportional hazards model mutually including all (dh)ceramides selected as direct effectors by the NetCoupler-algorithm (see methods section), further adjusting for total ceramide and total dihydro-ceramide concentrations, age in years (strata variable), sex, height, waist circumference, leisure-time physical activity, fasting status, antihypertensive medication, lipid-lowering medication, aspirin, total energy intake, smoking, alcohol consumption, educational attainment, plasma concentrations of triglycerides, total cholesterol, systolic and diastolic blood pressure, and HDL-cholesterol; baseline-prevalent T2D cases were excluded from the diabetes risk model, and adjusted for in the CVD risk model. P-values based on a two-sided likelihood ratio test.

Supplementary Table 10: Joint association of the selected (dh)ceramides with direct effects on CVD and T2D, excluding participants on lipid-lowering medication at baseline.

(dh)ceramide	HR (95%CI)	p-value
**CVD**		
dhCer22:2	1.57 (1.24, 1.99)	1.8E-04
Cer16:0	1.47 (1.1, 1.96)	0.0088
**T2D**		
Cer18:0	1.92 (1.36, 2.71)	1.9E-04
dhCer20:0	1.24 (1.01, 1.53)	0.0413
Cer22:0	1.89 (1.12, 3.19)	0.0171
dhCer26:1	0.88 (0.76, 1.02)	0.0921
Cer20:0	0.57 (0.36, 0.89)	0.0148
dhCer22:2	1.23 (0.99, 1.52)	0.0647

Hazard ratio (HR) per one standard deviation higher plasma concentration from the EPIC-Potsdam cohort, restricted to members of the nested case-cohort without lipid-lowering medication (CVD: n=1071 non-cases, and n=513 incident CVD cases; T2D: n=1056 non-cases, and n=694 incident T2D cases).

Risk estimates are from a model that mutually included all ceramides selected as direct effectors by the NetCoupler-algorithm (see methods section), further adjusting for total ceramide and total dihydro-ceramide concentrations, age in years (strata variable), sex, height, waist circumference, leisure-time physical activity, fasting status, antihypertensive medication, lipid-lowering medication, aspirin, total energy intake, smoking, alcohol consumption, educational attainment, plasma concentrations of triglycerides, total cholesterol, systolic and diastolic blood pressure, and HDL-cholesterol; baseline-prevalent T2D cases were excluded from the diabetes risk model, and adjusted for in the CVD risk model. P-values based on a two-sided likelihood ratio test

Supplementary Table 11: Joint association of the selected (dh)ceramides with direct effects on CVD and T2D, excluding participants with CVD or T2D incidence within the first two years of follow-up.

(dh)ceramide	HR (95% CI)	p-value
**CVD**		
dhCer22:2	1.59 (1.25, 2.03)	1.8E-04
Cer16:0	1.48 (1.09, 2)	0.0114
**T2D**		
Cer18:0	1.82 (1.29, 2.57)	6.6E-04
dhCer20:0	1.06 (0.87, 1.3)	0.5437
Cer22:0	1.67 (0.97, 2.87)	0.0636
dhCer26:1	0.9 (0.77, 1.04)	0.1589
Cer20:0	0.66 (0.41, 1.05)	0.0826
dhCer22:2	1.33 (1.06, 1.67)	0.0126

Hazard ratio (HR) per one standard deviation higher plasma concentration from the EPIC-Potsdam cohort, restricted to members of the nested case-cohort with two or more years of disease-free follow-up (CVD: n=1115 non-cases, and n=480 incident CVD cases, T2D: n=1096 non-cases, and n=574 incident T2D cases).

Risk estimates are from a model that mutually included all ceramides selected as direct effectors by the NetCoupler-algorithm (see methods section), further adjusting for total ceramide and total dihydro-ceramide concentrations, age in years (strata variable), sex, height, waist circumference, leisure-time physical activity, fasting status, antihypertensive medication, lipid-lowering medication, aspirin, total energy intake, smoking, alcohol consumption, educational attainment, plasma concentrations of triglycerides, total cholesterol, and systolic and diastolic blood pressure; baseline-prevalent T2D cases were excluded from the diabetes risk model, and adjusted for in the CVD risk model. All incident endpoints that occurred within the first two years of follow-up were excluded from the analysis. P-values based on a two-sided likelihood ratio test

Supplementary Table 12: Matching pathway names in Supplementary Figure 3 to the original database

Database	Pathway Name in Database	Pathway Name in Supplementary Figure 3	Category*
PID	AURORA A PATHWAY	AURORA A PATHWAY	cell cycle control
REACTOME	NCAM1 INTERACTIONS	NCAM1 INTERACTIONS	Developmental Biology
PID	RAC1 REG PATHWAY	RAC1 REG PATHWAY	Developmental Biology
REACTOME	SEMA3A PAK DEPENDENT AXON REPULSION	SEMA3A PAK DEPENDENT AXON REPULSION	Developmental Biology
REACTOME	SEMA3A PLEXIN REPULSION SIGNALING BY INHIBITING INTEGRIN ADHESION	SEMA3A PLEXIN REPULSION SIGNALING BY INHIBITING INTEGRIN ADHESION	Developmental Biology
REACTOME	DSCAM INTERACTIONS	DSCAM INTERACTIONS	Developmental Biology
NABA	COLLAGENS	COLLAGENS	Extracellular Matrix
NABA	ECM GLYCOPROTEINS	ECM GLYCOPROTEINS	Extracellular Matrix
PID	UPA UPAR PATHWAY	UROKINASE-TYPE PLASMINOGEN ACTIVATOR-MEDIATED SIGNALING	Hemostasis
REACTOME	GPVI MEDIATED ACTIVATION CASCADE	GPVI MEDIATED ACTIVATION CASCADE	Hemostasis
BIOCARTA	INTRINSIC PATHWAY	INTRINSIC PATHWAY	Hemostasis
REACTOME	PLATELET HOMEOSTASIS	PLATELET HOMEOSTASIS	Hemostasis
PID	THROMBIN PAR1 PATHWAY	THROMBIN SIGNALLING THROUGH PROTEINASE ACTIVATED RECEPTORS	Hemostasis
BIOCARTA	FIBRINOLYSIS PATHWAY	FIBRINOLYSIS PATHWAY	Hemostasis
REACTOME	NITRIC OXIDE STIMULATES GUANYLATE CYCLASE	NITRIC OXIDE STIMULATES GUANYLATE CYCLASE	Hemostasis
PID	INTEGRIN4 PATHWAY	BETA4 INTEGRIN CELL SURFACE INTERACTIONS	Hemostasis / Signal Transduction
PID	INTEGRIN1 PATHWAY	BETA1 INTEGRIN CELL SURFACE INTERACTIONS	Hemostasis / Signal Transduction
REACTOME	EFFECTS OF PIP2 HYDROLYSIS	EFFECTS OF PIP2 HYDROLYSIS	Hemostasis / Signal Transduction
PID	ILK PATHWAY	INTEGRIN-LINKED KINASE SIGNALING	Hemostasis / Signal Transduction
PID	INTEGRIN3 PATHWAY	INTEGRIN3 PATHWAY	Hemostasis
REACTOME	P130CAS LINKAGE TO MAPK SIGNALING FOR INTEGRINS	P130CAS LINKAGE TO MAPK SIGNALING FOR INTEGRINS	Hemostasis
BIOCARTA	LYM PATHWAY	ADHESION AND DIAPEDESIS OF LYMPHOCYTES	Immune System
KEGG	FOCAL ADHESION	FOCAL ADHESION	Immune System
KEGG	INTESTINAL IMMUNE NETWORK FOR IGA PRODUCTION	INTESTINAL IMMUNE NETWORK FOR IGA PRODUCTION	Immune system
REACTOME	CD28 DEPENDENT VAV1 PATHWAY	CD28 DEPENDENT VAV1 PATHWAY	Immune System
KEGG	FC GAMMA R MEDIATED PHAGOCYTOSIS	FC GAMMA R MEDIATED PHAGOCYTOSIS	Immune system
REACTOME	GENERATION OF SECOND	GENERATION OF SECOND MESSENGER	Immune

Database	Pathway Name in Database	Pathway Name in Supplementary Figure 3	Category*
	MESSENGER MOLECULES	MOLECULES	System
BIOCARTA	IL10 PATHWAY	IL10 PATHWAY	Immune system
PID	IL8 CXCR1 PATHWAY	IL8 CXCR1 PATHWAY	Immune system
PID	RAS PATHWAY	REGULATION OF RAS FAMILY ACTIVATION	Immune System
PID	TCR PATHWAY	T CELL ANTIGEN RECEPTOR (TCR) SIGNALING IN NAÏVE CD4+ T CELL	Immune system
REACTOME	TCR SIGNALING	T CELL ANTIGEN RECEPTOR (TCR) SIGNALING	Immune System
PID	TXA2PATHWAY	THROMBOXANE A2 RECEPTOR SIGNALING	Immune system
REACTOME	ANTIGEN PRESENTATION FOLDING ASSEMBLY AND PEPTIDE LOADING OF CLASS I MHC	ANTIGEN PRESENTATION FOLDING ASSEMBLY AND PEPTIDE LOADING OF CLASS I MHC	Immune System
REACTOME	INTEGRATION OF ENERGY METABOLISM	INTEGRATION OF ENERGY METABOLISM	Metabolism
REACTOME	LIPOPROTEIN METABOLISM	LIPOPROTEIN METABOLISM	Metabolism
REACTOME	ETHANOL OXIDATION	ETHANOL OXIDATION	Metabolism
KEGG	ASCORBATE AND ALDARATE METABOLISM	ASCORBATE AND ALDARATE METABOLISM	Metabolism
REACTOME	CHYLOMICRON MEDIATED LIPID TRANSPORT	CHYLOMICRON MEDIATED LIPID TRANSPORT	Metabolism
KEGG	DRUG METABOLISM OTHER ENZYMES	DRUG METABOLISM OTHER ENZYMES	Metabolism
KEGG	GLUTATHIONE METABOLISM	GLUTATHIONE METABOLISM	Metabolism
REACTOME	HDL MEDIATED LIPID TRANSPORT	HDL MEDIATED LIPID TRANSPORT	Metabolism
REACTOME	HEPARAN SULFATE HEPARIN HS GAG METABOLISM	HEPARAN SULFATE HEPARIN HS GAG METABOLISM	Metabolism
REACTOME	HS GAG BIOSYNTHESIS	HS GAG BIOSYNTHESIS	Metabolism
REACTOME	METABOLISM OF NUCLEOTIDES	METABOLISM OF NUCLEOTIDES	Metabolism
KEGG	METABOLISM OF XENOBIOTICS BY CYTOCHROME P450	METABOLISM OF XENOBIOTICS BY CYTOCHROME P450	Metabolism
KEGG	PYRIMIDINE METABOLISM	PYRIMIDINE METABOLISM	Metabolism
REACTOME	DOPAMINE NEUROTRANSMITTER RELEASE CYCLE	DOPAMINE NEUROTRANSMITTER RELEASE CYCLE	Neuronal System
REACTOME	NEUROTRANSMITTER RELEASE CYCLE	NEUROTRANSMITTER RELEASE CYCLE	Neuronal System
KEGG	CELL ADHESION MOLECULES CAMS	CELL ADHESION MOLECULES (CAMS)	Signal Transduction
PID	CXCR4 PATHWAY	CXCR4 PATHWAY	Signal Transduction
REACTOME	G ALPHA Z SIGNALLING EVENTS	G ALPHA Z SIGNALLING EVENTS	Signal Transduction
REACTOME	OPIOID SIGNALLING	OPIOID SIGNALLING	Signal Transduction
REACTOME	SIGNALING BY FGFR IN DISEASE	SIGNALING BY FGFR IN DISEASE	Signal transduction
KEGG	ECM RECEPTOR INTERACTION	ECM RECEPTOR INTERACTION	Signal Transduction
REACTOME	DOWNSTREAM SIGNALING OF ACTIVATED FGFR	DOWNSTREAM SIGNALING OF ACTIVATED FGFR	Signal Transduction
REACTOME	NGF SIGNALLING VIA TRKA FROM THE PLASMA MEMBRANE	NGF SIGNALLING VIA TRKA FROM THE PLASMA MEMBRANE	Signal Transduction
REACTOME	SIGNALING BY PDGF	SIGNALING BY PLATELET-DERIVED GROWTH FACTOR (PDGF)	Signal Transduction

Database	Pathway Name in Database	Pathway Name in Supplementary Figure 3	Category*
KEGG	ABC TRANSPORTERS	ABC TRANSPORTERS	Transport of small molecules
REACTOME	ORGANIC CATION ANION ZWITTERION TRANSPORT	ORGANIC CATION ANION ZWITTERION TRANSPORT	Transport of small molecules
KEGG	GAP JUNCTION	GAP JUNCTION	Vesicle-mediated transport
REACTOME	MUSCLE CONTRACTION	MUSCLE CONTRACTION	Muscle contraction

*Pathway categories from REACTOME, modified.

Supplementary Figures

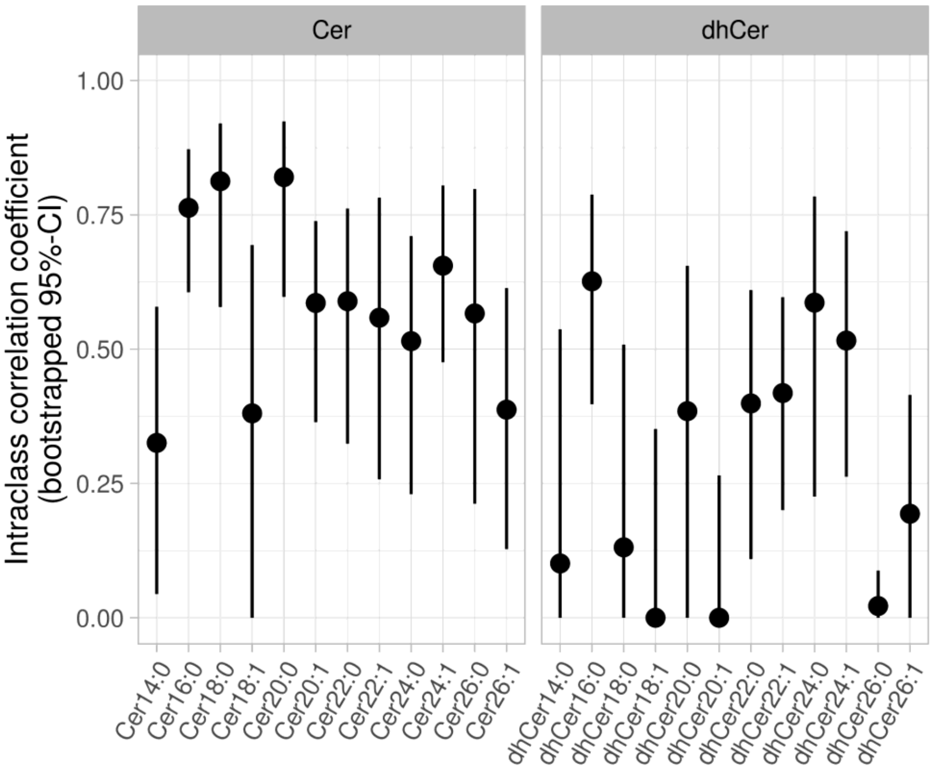

Supplementary Figure 1: Biological reliability of repeated ceramide and dihydroceramide measurements.

Dots mark median and lines the 95% confidence interval from a bootstrapping procedure with 1000 replicates.

	Cer14:0	Cer16:0	Cer18:0	Cer18:1	Cer20:0	Cer20:1	Cer22:0	Cer22:1	Cer24:0	Cer24:1	Cer26:0	Cer26:1	dhCer14:0	dhCer16:0	dhCer18:0	dhCer18:1	dhCer20:0	dhCer20:1	dhCer22:0	dhCer22:1	dhCer22:2	dhCer24:0	dhCer24:1	dhCer26:0	dhCer26:1
Cer14:0		0.24	0.00	0.15	0.10	0.01	-0.07	0.09	0.12	-0.24	0.19	0.10	0.11	-0.02	-0.05	0.07	0.04	0.05	-0.02	-0.01	0.08	-0.11	0.03	-0.15	0.12
Cer16:0	0.56		0.24	0.13	-0.01	0.02	0.14	0.07	0.03	0.07	-0.03	0.08	-0.02	0.54	-0.10	-0.09	-0.05	-0.14	-0.03	0.00	-0.05	0.00	-0.08	0.03	0.01
Cer18:0	0.43	0.73		0.05	0.61	-0.12	-0.02	-0.08	-0.19	0.05	0.01	0.03	-0.07	-0.13	0.37	0.01	-0.03	-0.04	-0.01	-0.02	0.09	0.11	0.01	0.01	0.01
Cer18:1	0.61	0.56	0.51		0.08	0.19	-0.09	0.11	0.10	-0.01	0.05	-0.08	0.08	0.08	0.07	0.16	0.01	0.10	0.04	0.01	-0.09	-0.12	0.01	-0.08	0.01
Cer20:0	0.47	0.76	0.88	0.54		0.10	0.37	-0.08	-0.06	0.26	-0.02	-0.07	-0.03	-0.04	-0.06	-0.01	0.16	0.00	-0.05	-0.05	-0.11	-0.03	0.03	0.02	0.00
Cer20:1	0.41	0.31	0.27	0.57	0.34		-0.01	0.32	-0.02	-0.06	0.15	-0.02	0.11	-0.09	0.14	0.03	-0.03	0.09	0.05	0.09	-0.10	-0.05	0.03	-0.11	-0.07
Cer22:0	0.45	0.77	0.70	0.47	0.84	0.31		0.40	0.56	0.15	-0.02	-0.30	-0.04	-0.07	-0.09	-0.01	-0.14	0.05	0.45	0.00	0.00	-0.12	-0.12	0.01	0.00
Cer22:1	0.40	0.58	0.48	0.47	0.57	0.51	0.63		-0.30	0.11	-0.07	0.21	-0.15	0.00	-0.01	-0.06	0.01	-0.09	0.00	0.19	0.00	0.04	-0.02	0.10	0.06
Cer24:0	0.46	0.70	0.57	0.40	0.70	0.19	0.89	0.43		0.16	0.16	0.08	0.10	-0.06	-0.05	0.03	0.11	-0.05	-0.25	0.05	0.10	0.72	-0.13	-0.14	0.01
Cer24:1	0.38	0.72	0.74	0.44	0.82	0.27	0.79	0.58	0.74		0.00	0.49	0.11	0.01	0.00	0.02	0.00	0.03	-0.14	-0.06	0.06	-0.17	0.36	-0.08	-0.18
Cer26:0	0.42	0.41	0.31	0.30	0.35	0.25	0.40	0.30	0.48	0.36		0.20	-0.03	0.01	0.07	0.01	-0.07	-0.11	-0.02	0.02	-0.07	0.04	-0.11	0.69	0.12
Cer26:1	0.36	0.54	0.49	0.28	0.50	0.17	0.48	0.45	0.55	0.72	0.42		-0.05	0.01	-0.02	-0.04	-0.03	0.04	0.01	-0.04	0.09	0.10	0.03	-0.13	0.06
dhCer14:0	0.49	0.26	0.23	0.56	0.26	0.44	0.26	0.18	0.29	0.26	0.23	0.16		0.07	0.07	0.21	0.14	0.21	0.05	0.06	0.02	-0.11	-0.07	0.08	0.05
dhCer16:0	0.54	0.75	0.55	0.60	0.57	0.35	0.59	0.45	0.57	0.56	0.38	0.44	0.48		0.23	0.07	0.07	0.09	0.17	-0.06	-0.03	0.11	0.01	0.04	-0.04
dhCer18:0	0.47	0.51	0.63	0.61	0.56	0.44	0.46	0.36	0.43	0.50	0.32	0.36	0.54	0.66		0.08	0.15	0.11	0.05	-0.06	0.05	0.10	0.04	-0.05	-0.07
dhCer18:1	0.51	0.28	0.30	0.61	0.32	0.44	0.30	0.23	0.32	0.30	0.25	0.19	0.73	0.51	0.59		0.09	0.28	0.00	0.04	0.13	-0.02	-0.06	0.00	0.12
dhCer20:0	0.52	0.44	0.47	0.60	0.51	0.44	0.47	0.39	0.45	0.46	0.31	0.31	0.66	0.60	0.67	0.68		0.11	0.17	0.26	-0.06	-0.06	0.10	0.09	0.00
dhCer20:1	0.46	0.24	0.27	0.57	0.29	0.45	0.28	0.23	0.27	0.28	0.17	0.18	0.73	0.49	0.59	0.76	0.68		0.05	0.12	0.18	-0.01	-0.08	0.04	0.01
dhCer22:0	0.51	0.68	0.62	0.56	0.71	0.41	0.82	0.58	0.75	0.67	0.39	0.45	0.45	0.71	0.62	0.50	0.67	0.50		0.13	0.08	0.32	0.20	-0.04	-0.08
dhCer22:1	0.45	0.29	0.28	0.51	0.32	0.48	0.35	0.44	0.30	0.32	0.28	0.23	0.58	0.43	0.48	0.60	0.70	0.63	0.56		0.19	-0.14	0.17	0.04	0.11
dhCer22:2	0.45	0.42	0.44	0.41	0.46	0.26	0.53	0.35	0.57	0.54	0.31	0.45	0.49	0.51	0.53	0.56	0.58	0.57	0.65	0.58		0.03	0.15	0.04	-0.06
dhCer24:0	0.41	0.68	0.59	0.36	0.67	0.17	0.83	0.43	0.93	0.71	0.49	0.57	0.24	0.61	0.47	0.30	0.45	0.26	0.78	0.29	0.58		0.21	0.01	0.02
dhCer24:1	0.44	0.53	0.57	0.45	0.60	0.32	0.58	0.50	0.57	0.68	0.39	0.56	0.38	0.56	0.56	0.44	0.62	0.42	0.71	0.58	0.64	0.63		0.13	0.47
dhCer26:0	0.14	0.09	0.07	0.10	0.06	0.12	0.05	0.14	0.06	0.04	0.63	0.08	0.18	0.17	0.16	0.17	0.23	0.14	0.14	0.26	0.15	0.11	0.25		0.01
dhCer26:1	0.44	0.27	0.26	0.35	0.27	0.24	0.28	0.31	0.31	0.28	0.39	0.32	0.37	0.34	0.33	0.42	0.46	0.36	0.41	0.51	0.42	0.35	0.63	0.31	

Supplementary Figure 2: Correlations (lower left triangle) and partial correlations (upper left triangle) among (dh)ceramides.

Partial correlations are conditional on all other (dh)ceramides.

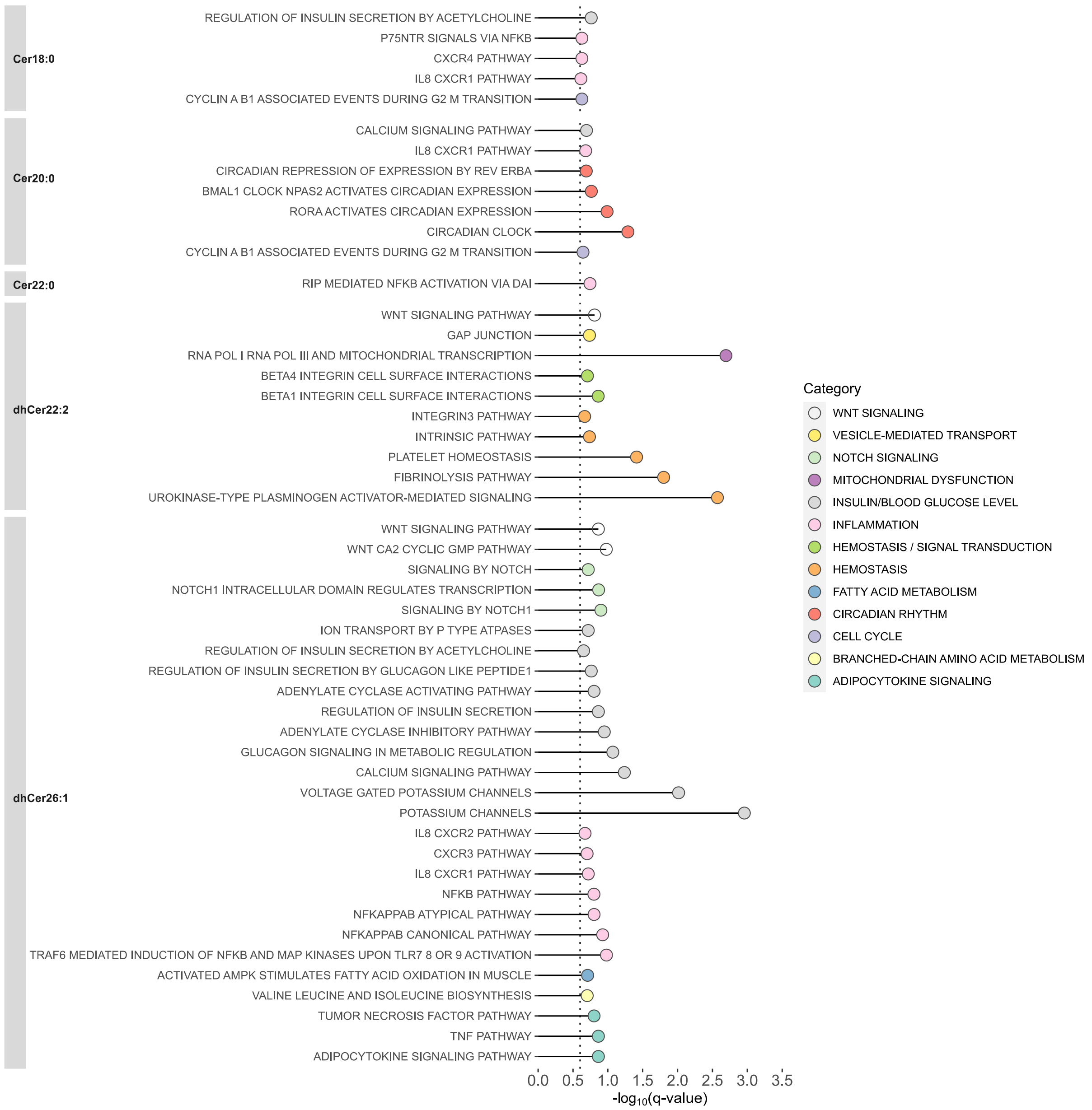

Supplementary Figure 3: Pathway enrichment analysis.

Shown are pathways with significant enrichment of SNPs associated with i) the disease endpoint and ii) with the disease-associated ceramide. Colored frames around grey ceramide-named strip encode the ceramide disease-association: green – type 2 diabetes; purple – cardiovascular disease; brown – both endpoints.

Additional description of Supplementary Figure 3:

Among the (dh)ceramides linked to both T2D and CVD, Cer22:0-associated SNPs were enriched in gene sets linked to NF-kappa B activation and the Interleukin 10-pathway. For dhCer22:2, SNPs were enriched in multiple pathways that have a decisive role in membrane signaling in common: mitochondrial function, hemostasis & platelet signaling, cell-cell interactions, adaptive immune responses including adhesion and diapedesis of lymphocytes, signal transduction, regulation of insulin secretion, and regulation of other metabolic processes.
Among the only T2D-related (dh)ceramides, we detected that Cer16:0-, Cer18:0-, and Cer26:0-associated SNPs were enriched in insulin regulatory pathways. Moreover, Cer18:0 and Cer26:0 had enriched genetic associations in cytokine signaling- and inflammation-pathways (e.g., Interleukin- and NFkappaB-

signaling). Genetic signals on dhCer20:0 were enriched in glycolysis, gluconeogenesis, and in fatty acid metabolism pathway-related genes. GWAS-results for Cer20:0 were not enriched in T2D-related pathways.
Among only CVD-related (dh)ceramides, genetic signals on Cer14:0 were enriched in lipoprotein metabolism and muscle contraction related gene sets. For Cer22:1, we detected enriched genetic signals in multiple pathways, most of which were linked to cell-cell interactions, hemostasis & platelet signaling, and adaptive immune response; moreover, this metabolite was genetically linked to signal transduction, transmembrane transport, and regulation of intermediary metabolism. GWAS-signals on dhCer24:0 were not enriched in CVD-related pathways.

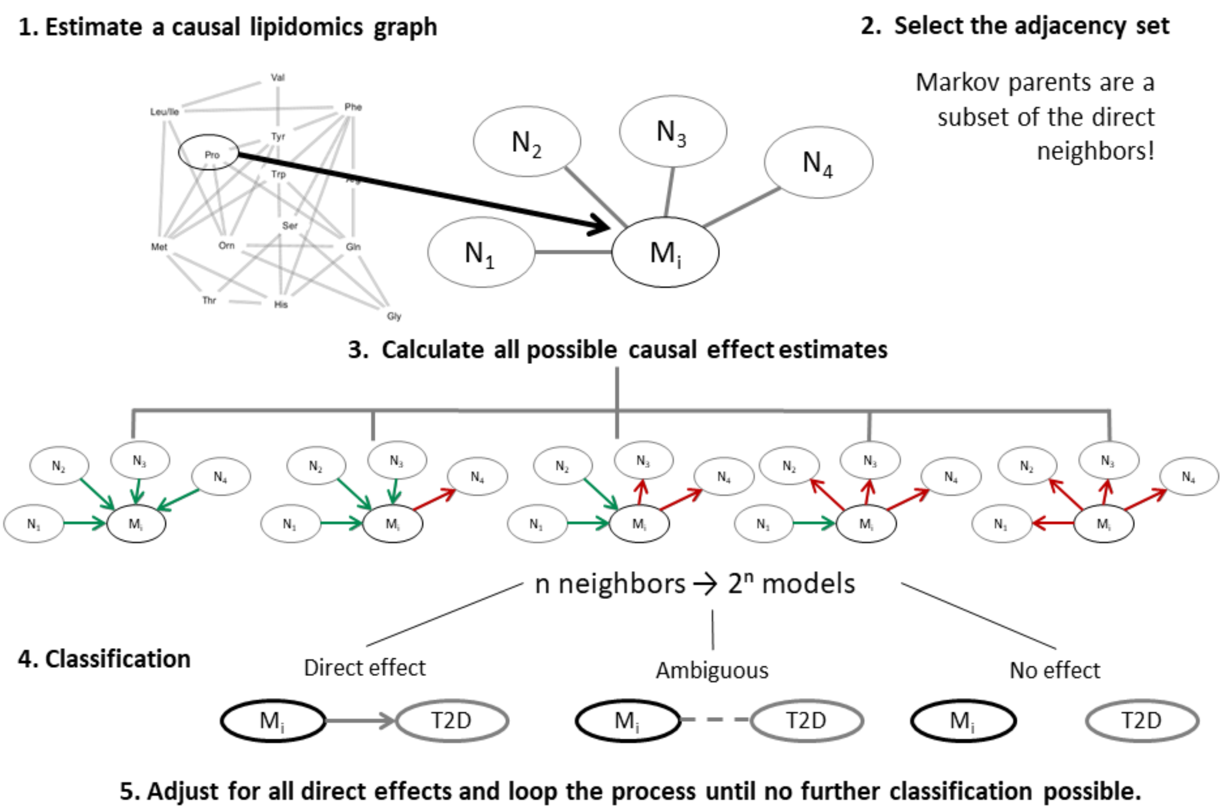

Supplementary Figure 4: The NetCoupler algorithm.
