# Supplementary material for: Dihydroceramide- and ceramide-profiling provides insights into human cardiometabolic disease etiology
Source: Nat Commun. 2022 Feb 17;13:936. doi: 10.1038/s41467-022-28496-1 (PMC8854598; doi:10.1038/s41467-022-28496-1)
Supplement: Supplementary file 3 — Description of Additional Supplementary Files [file 41467_2022_28496_MOESM3_ESM.docx]

**Supplementary Data captions**

**Supplementary Data 1: Lookup-up of EPIC-Potsdam GWAS signals on ceramides C18:0, C20:0, and C22:0 in EUROSPAN.**

Single nucleotide polymorphisms that were associated with type 2 diabetes-associated ceramides C16:0, C18:0, and C22:0 at a genome-wide suggestive statistical significance level (p-value < 10^-5^) in a subsample of the EPIC-Potsdam study with GWAS and lipidomics data and available for lookup in the EUROSPAN consortium. Dihydro-ceramides and other disease-related cermides from EPIC Potsdam were not available in EUROSPAN. Abbreviations: Rsid, RSID; Chrom.:position, chromosome and position; Nearest Gene(s), Nearby gene(s); Gene ID (Ensemble), Ensemble IDs of nearby gene(s); beta, beta estimate from a frequentist additive model; SE, standard error from a frequentist additive model; p-value, p-value from a frequentist additive model; Crom.:Pos., Crhomosome:Position; A(ref.), Allele A (reference); B(coding); Allele B (coding); AA (n), Major allele homozygote count; AB (n), Heterozygote count; BB (n), minor allele homozygote count; MAF, minor allele frequency.

**Supplementary Data 2: GWAS signals on ceramide C16:0.**

Single nucleotide polymorphisms that were associated with cardiovascular disease-associated ceramide C16:0 at a genome-wide suggestive statistical significance level (p-value < 10^-5^) in a subsample of the EPIC-Potsdam study with GWAS and lipidomics data. Abbreviations: Rsid, RSID; Chrom.:position, chromosome and position; Nearest Gene(s), Nearby gene(s); Gene ID (Ensemble), Ensemble IDs of nearby gene(s); beta, beta estimate from a frequentist additive model; SE, standard error from a frequentist additive model; p-value, p-value from a frequentist additive model; Crom.:Pos., Crhomosome:Position; A(ref.), Allele A (reference); B(coding); Allele B (coding); AA (n), Major allele homozygote count; AB (n), Heterozygote count; BB (n), minor allele homozygote count; MAF, minor allele frequency.

**Supplementary Data 3: GWAS signals on ceramide C18:0.**

Single nucleotide polymorphisms that were associated with type 2 diabetes-associated ceramide C18:0 at a genome-wide suggestive statistical significance level (p-value < 10^-5^) in a subsample of the EPIC-Potsdam study with GWAS and lipidomics data. Abbreviations: Rsid, RSID; Chrom.:position, chromosome and position; Nearest Gene(s), Nearby gene(s); Gene ID (Ensemble), Ensemble IDs of nearby gene(s); beta, beta estimate from a frequentist additive model; SE, standard error from a frequentist additive model; p-value, p-value from a frequentist additive model; Crom.:Pos., Crhomosome:Position; A(ref.), Allele A (reference); B(coding); Allele B (coding); AA (n), Major allele homozygote count; AB (n), Heterozygote count; BB (n), minor allele homozygote count; MAF, minor allele frequency.

**Supplementary Data 4: GWAS signals on ceramide C20:0.**

Single nucleotide polymorphisms that were associated with type 2 diabetes-associated **ceramide C20:0** at a genome-wide suggestive statistical significance level (p-value < 10^-5^) in a subsample of the EPIC-Potsdam study with GWAS and lipidomics data. Abbreviations: Rsid, RSID; Chrom.:position, chromosome and position; Nearest Gene(s), Nearby gene(s); Gene ID (Ensemble), Ensemble IDs of nearby gene(s); beta, beta estimate from a frequentist additive model; SE, standard error from a frequentist additive model; p-value, p-value from a frequentist additive model; Crom.:Pos., Crhomosome:Position; A(ref.), Allele A (reference); B(coding); Allele B (coding); AA (n), Major allele homozygote count; AB (n), Heterozygote count; BB (n), minor allele homozygote count; MAF, minor allele frequency.

**Supplementary Data 5: GWAS signals on ceramide C22:0.**

Single nucleotide polymorphisms that were associated with type 2 diabetes-associated **ceramide C22:0** at a genome-wide suggestive statistical significance level (p-value < 10^-5^) in a subsample of the EPIC-Potsdam study with GWAS and lipidomics data. Abbreviations: Rsid, RSID; Chrom.:position, chromosome and position; Nearest Gene(s), Nearby gene(s); Gene ID (Ensemble), Ensemble IDs of nearby gene(s); beta, beta estimate from a frequentist additive model; SE, standard error from a frequentist additive model; p-value, p-value from a frequentist additive model; Crom.:Pos., Crhomosome:Position; A(ref.), Allele A (reference); B(coding); Allele B (coding); AA (n), Major allele homozygote count; AB (n), Heterozygote count; BB (n), minor allele homozygote count; MAF, minor allele frequency.

**Supplementary Data 6: GWAS signals on dihydro-ceramide C20:0.**

Single nucleotide polymorphisms that were associated with type 2 diabetes-associated dihydro-ceramide C20:0 at a genome-wide suggestive statistical significance level (p-value < 10^-5^) in a subsample of the EPIC-Potsdam study with GWAS and lipidomics data. Abbreviations: Rsid, RSID; Chrom.:position, chromosome and position; Nearest Gene(s), Nearby gene(s); Gene ID (Ensemble), Ensemble IDs of nearby gene(s); beta, beta estimate from a frequentist additive model; SE, standard error from a frequentist additive model; p-value, p-value from a frequentist additive model; Crom.:Pos., Crhomosome:Position; A(ref.), Allele A (reference); B(coding); Allele B (coding); AA (n), Major allele homozygote count; AB (n), Heterozygote count; BB (n), minor allele homozygote count; MAF, minor allele frequency.

**Supplementary Data 7: GWAS signals on dihydro-ceramide C22:2.**

Single nucleotide polymorphisms that were associated with cardiovascular disease- and type 2 diabetes-associated dihydro-ceramide C22:2 at a genome-wide suggestive statistical significance level (p-value < 10^-5^) in a subsample of the EPIC-Potsdam study with GWAS and lipidomics data.Abbreviations: Rsid, RSID; Chrom.:position, chromosome and position; Nearest Gene(s), Nearby gene(s); Gene ID (Ensemble), Ensemble IDs of nearby gene(s); beta, beta estimate from a frequentist additive model; SE, standard error from a frequentist additive model; p-value, p-value from a frequentist additive model; Crom.:Pos., Crhomosome:Position; A(ref.), Allele A (reference); B(coding); Allele B (coding); AA (n), Major allele homozygote count; AB (n), Heterozygote count; BB (n), minor allele homozygote count; MAF, minor allele frequency.

**Supplementary Data 8: GWAS signals on dihydro-ceramide C26:1.**

Single nucleotide polymorphisms that were associated with type 2 diabetes-associated dihydro-ceramide C26:1 at a genome-wide suggestive statistical significance level (p-value < 10^-5^) in a subsample of the EPIC-Potsdam study with GWAS and lipidomics data. Abbreviations: Rsid, RSID; Chrom.:position, chromosome and position; Nearest Gene(s), Nearby gene(s); Gene ID (Ensemble), Ensemble IDs of nearby gene(s); beta, beta estimate from a frequentist additive model; SE, standard error from a frequentist additive model; p-value, p-value from a frequentist additive model; Crom.:Pos., Crhomosome:Position; A(ref.), Allele A (reference); B(coding); Allele B (coding); AA (n), Major allele homozygote count; AB (n), Heterozygote count; BB (n), minor allele homozygote count; MAF, minor allele frequency.

**Supplementary Data 9: Identifying information for the analyzed ceramides and dihydroceramides.**

The Human Metabolome Database (HMDB) is a freely available electronic database containing detailed information about small molecule metabolites. LIPID MAPS is a web portal designed to be a gateway to Lipidomics resources. ID: unique identifier.
