# Supplementary material for: Dihydroceramide- and ceramide-profiling provides insights into human cardiometabolic disease etiology
Source: Nat Commun. 2022 Feb 17;13:936. doi: 10.1038/s41467-022-28496-1 (PMC8854598; doi:10.1038/s41467-022-28496-1)
Supplement: Supplementary file 13 — Reporting summary [file 41467_2022_28496_MOESM13_ESM.pdf]

nature research

Corresponding author(s): Matthias B Schulze

Last updated by author(s): Dec 2, 2021

Reporting Summary

Nature Research wishes to improve the reproducibility of the work that we publish. This form provides structure for consistency and transparency in reporting. For further information on Nature Research policies, see our Editorial Policies and the Editorial Policy Checklist.

Statistics

For all statistical analyses, confirm that the following items are present in the figure legend, table legend, main text, or Methods section.

n/a	Confirmed	
☐	☒	The exact sample size (n) for each experimental group/condition, given as a discrete number and unit of measurement
☐	☒	A statement on whether measurements were taken from distinct samples or whether the same sample was measured repeatedly
☐	☒	The statistical test(s) used AND whether they are one- or two-sided<br>*Only common tests should be described solely by name; describe more complex techniques in the Methods section.*
☐	☒	A description of all covariates tested
☐	☒	A description of any assumptions or corrections, such as tests of normality and adjustment for multiple comparisons
☐	☒	A full description of the statistical parameters including central tendency (e.g. means) or other basic estimates (e.g. regression coefficient) AND variation (e.g. standard deviation) or associated estimates of uncertainty (e.g. confidence intervals)
☐	☒	For null hypothesis testing, the test statistic (e.g. F, t, r) with confidence intervals, effect sizes, degrees of freedom and P value noted<br>*Give P values as exact values whenever suitable.*
☒	☐	For Bayesian analysis, information on the choice of priors and Markov chain Monte Carlo settings
☐	☒	For hierarchical and complex designs, identification of the appropriate level for tests and full reporting of outcomes
☐	☒	Estimates of effect sizes (e.g. Cohen's d, Pearson's r), indicating how they were calculated

Our web collection on statistics for biologists contains articles on many of the points above.

Software and code

Policy information about availability of computer code

Data collection

FoxPro version 2.6 was used for EPIC Potsdam baseline data collection.

Data analysis

In EPIC-Potsdam, the Statistical Analysis System (SAS) Enterprise Guide 7.1 with SAS version 9.4 (SAS Institute Inc., Cary, NC, USA) was used to manage and prepare datasets and transform the lipid values. The pcalg-package and the NetCoupler-package in R (modified custom code based on version 3.5.2 (2018-12-20), https://github.com/NetCoupler) were used to generate the (dh)ceramide network and link it to disease incidences. The developmentNetCoupler versions and settings used to generate the results herein are available on request from the corresponding author [MBS].
The software QCtool v1.4 and SNPtest v2.5.2 (1) were used for the GWAS on lipids. Variants were mapped to Ensembl annotation version 84 (GRCh37) (101), and we used the Ensembl Variant Effect Predictor for annotation (102). We used GSA-SNP2 software for gene set enrichment analysis based on GWAS p-values (2). We used pathway annotation from the MSigDB C2.CP (curated canonical pathways) version 5.2 database (3), therein the C2 canonical pathway database, which consists of 1329 curated gene sets that represent a biological process compiled by domain experts (4, 5).
For the Mendelian Randomization (MR) analyses, we used the R-packages 'TwoSampleMR’ (v0.5.5) from the MR-Base platform (6) and the “MendelianRandomization” (v0.5.0) (7) R packages R (version 3.6.3 (2020-02-29)). Mediation analyses were conducted in R (version 3.5.2 (2018-12-20)). Bias corrected 95% bootstrap confidence intervals for the proportion explained by mediators were constructed with the bcajack-function from the bcaboot package (CRAN.R-project.org/package=bcaboot) with 1000 replications and a two-thirds sampling fraction.
Genetic association tests between lipid and allele dosage in EUROSPAN were performed using a mixed model approach implemented with the 'mmscore' option in the GenABEL software, and results from the five populations were combined using inverse variance weighted fixed-effects model meta-analyses using the METAL software.

1. Marchini J, Howie B, Myers S, McVean G, Donnelly P. A new multipoint method for genome-wide association studies by imputation of genotypes. Nat Genet. 2007;39:906-913.

2. Yoon S, Nguyen Hai C T, Yoo YJ, Kim J, Baik B, Kim S, Kim J, Kim S, Nam D. Efficient pathway enrichment and network analysis of GWAS summary data using GSA-SNP2. Nucleic Acids Research. 2018;46:e60-e60.
3. Liberzon A, Subramanian A, Pinchback R, Thorvaldsdottir H, Tamayo P, Mesirov JP. Molecular signatures database (MSigDB) 3.0. Bioinformatics. 2011;27:1739-1740.
4. Huang Y, Yang J, Shen J, Chen FF, Yu Y. Sphingolipids are involved in N-methyl-N'-nitro-N-nitrosoguanidine-induced epidermal growth factor receptor clustering. Biochem Biophys Res Commun. 2005;330:430-438.
5. Subramanian A, Tamayo P, Mootha VK, et al. Gene set enrichment analysis: a knowledge-based approach for interpreting genome-wide expression profiles. Proceedings of the National Academy of Sciences of the United States of America. 2005;102:15545-15550.
6. Hemani G, Zheng J, Elsworth B, et al. The MR-Base platform supports systematic causal inference across the human phenome. Elife. 2018;7.
7. Yavorska OO, Burgess S. MendelianRandomization: an R package for performing Mendelian randomization analyses using summarized data. Int J Epidemiol. 2017;46:1734-1739.

For manuscripts utilizing custom algorithms or software that are central to the research but not yet described in published literature, software must be made available to editors and reviewers. We strongly encourage code deposition in a community repository (e.g. GitHub). See the Nature Research guidelines for submitting code & software for further information.

Data

Policy information about availability of data

All manuscripts must include a data availability statement. This statement should provide the following information, where applicable:

- Accession codes, unique identifiers, or web links for publicly available datasets
- A list of figures that have associated raw data
- A description of any restrictions on data availability

The data that support the findings of this study are not publicly available due to data protection regulations. In accordance with German Federal and State data protection regulations, epidemiological data analyses of EPIC-Potsdam may be initiated upon an informal inquiry addressed to the PI of the EPIC-Potsdam study, who is the corresponding author of this manuscript [MBS]. EPIC-Potsdam study data is stored (in SAS data files) and analyzed on servers of the German Institute of Human Nutrition Potsdam-Rehbruecke, and analyses requests are discussed in monthly meetings and usually approved when concerns regarding data privacy can be ruled out. Metabolite sets for enrichment analysis were obtained from the MSigDB C2.CP database together with GSA-SNP2 tool (https://sourceforge.net/projects/gsasnp2/files/data/popular_pathway_data-20170227T151601Z-001.zip).

Field-specific reporting

Please select the one below that is the best fit for your research. If you are not sure, read the appropriate sections before making your selection.

☒ Life sciences ☐ Behavioural & social sciences ☐ Ecological, evolutionary & environmental sciences

For a reference copy of the document with all sections, see nature.com/documents/nr-reporting-summary-flat.pdf

Life sciences study design

All studies must disclose on these points even when the disclosure is negative.

Sample size

The sample size does not rely primarily on power calculations. The observational analyses were based on two case-cohort samples nested within the prospective EPIC-Potsdam study (775 participants with incident T2D among 1886 at-risk participants, and 551 participants with incident CVD among 1707 at-risk participants). The sample size of the case cohort was determined by the number of incident disease cases in the EPIC-Potsdam cohort at the time of study construction. For T2D, the censoring date was the 31st of August 2005; for CVD the censoring date was the 30th of November 2006. The subcohort (n=1,137) included participants randomly selected from all participants with available baseline blood samples and free of T2D at baseline.

Data exclusions

For T2D, the censoring date was the 31st of August 2005 (820 incident cases). After excluding participants with missing follow-up information, prevalent diabetes at recruitment, insufficient blood specimens, or non-verifiable information on diabetes incidence, the analytical sample comprised 1886 participants (1000 women and 886 men), including 775 participants with incident T2D from whom 70 were part of the subcohort. The median follow-up time for T2D was 6.5 years (interquartile range 6.0 to 8.6 years).

For CVD, the censoring date was the 30th of November 2006, with 583 incident primary cardiovascular events occurring during the study. After equivalent exclusions (using prevalent and non-verifiable CVD instead of diabetes as exclusion criterion), the CVD sample comprised 1707 participants (910 women and 797 men), including 551 participants with incident CVD (283 only myocardial infarction, 257 only stroke, 11 both) from whom 30 were part of the subcohort. The median follow-up time for CVD was 8.4 years (interquartile range 7.6 to 9.2 years).

Replication

No experiments were performed. The observational associations between (dh)ceramides and cardiometabolic disease incidence were not externally validated.

For the EPIC-Potsdam GWAS on Cer18:0, Cer20:0, and Cer22:0, we performed lookup studies in two independent, first with partly unpublished results from EUROSPAN (European special populations research network: quantifying and harnessing genetic variation for gene discovery) consortium, and second with published SNP-Cer22:0 associations from the Framingham Heart Study Offspring Cohort (n=2217, Cer18:0 and Cer20:0 not available). The other (dh)ceramides associated with cardiometabolic risk in EPIC-Potsdam were not available in the Framingham Heart Study Offspring Cohort. These resources were also used to replicate Mendelian randomization analyses for Cer 22:0. No other replications were attempted.

	Associations of SNPs with other (dh)ceramides were not externally validated.
Randomization	N/A (observational analysis).
Blinding	The laboratory personnel that generated the (dh)ceramide measurements was blinded to the case status and all other phenotypical characteristics of the blood specimen donors. As usual, the epidemiological data analysts were not blinded.

Behavioural & social sciences study design

All studies must disclose on these points even when the disclosure is negative.

Study description	*Briefly describe the study type including whether data are quantitative, qualitative, or mixed-methods (e.g. qualitative cross-sectional, quantitative experimental, mixed-methods case study).*
Research sample	*State the research sample (e.g. Harvard university undergraduates, villagers in rural India) and provide relevant demographic information (e.g. age, sex) and indicate whether the sample is representative. Provide a rationale for the study sample chosen. For studies involving existing datasets, please describe the dataset and source.*
Sampling strategy	*Describe the sampling procedure (e.g. random, snowball, stratified, convenience). Describe the statistical methods that were used to predetermine sample size OR if no sample-size calculation was performed, describe how sample sizes were chosen and provide a rationale for why these sample sizes are sufficient. For qualitative data, please indicate whether data saturation was considered, and what criteria were used to decide that no further sampling was needed.*
Data collection	*Provide details about the data collection procedure, including the instruments or devices used to record the data (e.g. pen and paper, computer, eye tracker, video or audio equipment) whether anyone was present besides the participant(s) and the researcher, and whether the researcher was blind to experimental condition and/or the study hypothesis during data collection.*
Timing	*Indicate the start and stop dates of data collection. If there is a gap between collection periods, state the dates for each sample cohort.*
Data exclusions	*If no data were excluded from the analyses, state so OR if data were excluded, provide the exact number of exclusions and the rationale behind them, indicating whether exclusion criteria were pre-established.*
Non-participation	*State how many participants dropped out/declined participation and the reason(s) given OR provide response rate OR state that no participants dropped out/declined participation.*
Randomization	*If participants were not allocated into experimental groups, state so OR describe how participants were allocated to groups, and if allocation was not random, describe how covariates were controlled.*

Ecological, evolutionary & environmental sciences study design

All studies must disclose on these points even when the disclosure is negative.

Study description	*Briefly describe the study. For quantitative data include treatment factors and interactions, design structure (e.g. factorial, nested, hierarchical), nature and number of experimental units and replicates.*
Research sample	*Describe the research sample (e.g. a group of tagged Passer domesticus, all Stenocereus thurberi within Organ Pipe Cactus National Monument), and provide a rationale for the sample choice. When relevant, describe the organism taxa, source, sex, age range and any manipulations. State what population the sample is meant to represent when applicable. For studies involving existing datasets, describe the data and its source.*
Sampling strategy	*Note the sampling procedure. Describe the statistical methods that were used to predetermine sample size OR if no sample-size calculation was performed, describe how sample sizes were chosen and provide a rationale for why these sample sizes are sufficient.*
Data collection	*Describe the data collection procedure, including who recorded the data and how.*
Timing and spatial scale	*Indicate the start and stop dates of data collection, noting the frequency and periodicity of sampling and providing a rationale for these choices. If there is a gap between collection periods, state the dates for each sample cohort. Specify the spatial scale from which the data are taken*
Data exclusions	*If no data were excluded from the analyses, state so OR if data were excluded, describe the exclusions and the rationale behind them, indicating whether exclusion criteria were pre-established.*
Reproducibility	*Describe the measures taken to verify the reproducibility of experimental findings. For each experiment, note whether any attempts to repeat the experiment failed OR state that all attempts to repeat the experiment were successful.*
Randomization	*Describe how samples/organisms/participants were allocated into groups. If allocation was not random, describe how covariates were controlled. If this is not relevant to your study, explain why.*

Blinding | *Describe the extent of blinding used during data acquisition and analysis. If blinding was not possible, describe why OR explain why blinding was not relevant to your study.*

Did the study involve field work? ☐ Yes ☐ No

Field work, collection and transport

Field conditions | *Describe the study conditions for field work, providing relevant parameters (e.g. temperature, rainfall).*

Location | *State the location of the sampling or experiment, providing relevant parameters (e.g. latitude and longitude, elevation, water depth).*

Access & import/export | *Describe the efforts you have made to access habitats and to collect and import/export your samples in a responsible manner and in compliance with local, national and international laws, noting any permits that were obtained (give the name of the issuing authority, the date of issue, and any identifying information).*

Disturbance | *Describe any disturbance caused by the study and how it was minimized.*

Reporting for specific materials, systems and methods

We require information from authors about some types of materials, experimental systems and methods used in many studies. Here, indicate whether each material, system or method listed is relevant to your study. If you are not sure if a list item applies to your research, read the appropriate section before selecting a response.

Materials & experimental systems

n/a	Involved in the study	
☒	☐	Antibodies
☒	☐	Eukaryotic cell lines
☒	☐	Palaeontology and archaeology
☒	☐	Animals and other organisms
☐	☒	Human research participants
☒	☐	Clinical data
☒	☐	Dual use research of concern

Methods

n/a	Involved in the study	
☒	☐	ChIP-seq
☒	☐	Flow cytometry
☒	☐	MRI-based neuroimaging

Human research participants

Policy information about studies involving human research participants

Population characteristics | The prospective EPIC-Potsdam cohort study includes 27,548 participants (16,644 women and 10,904 men) recruited within an age-range of 35-65 years from the general population between 1994 and 1998. All participants were Whites of Middle European descent. The participants in the analyses sample for incident T2D were free of prevalent T2D and CVD at baseline; the participants in the analyses sample for incident CVD were free of prevalent CVD at baseline

Recruitment | The prospective EPIC-Potsdam cohort study recruited 27,548 participants (16,644 women and 10,904 men) within an age-range of 35-65 years from the general population between 1994 and 1998. Participants were then actively contacted by sending out questionnaires and, if necessary, by telephone every 2-3 years. Compared to the general population in the Potsdam area, EPIC-Potsdam participants tended have higher education, a healthier lifestyle, and were more likely to be women. However, valid etiological research in population-based epidemiological cohort studies does not depend on representative population samples. The primary recruitment-related source of potential bias is loss to follow-up. The EPIC-Potsdam study had very low loss to follow-up with response rates between 90% and 96% in the included follow-up rounds.

Ethics oversight | All EPIC-Potsdam participants gave informed consent for biomedical research use of their data, and the study was approved by the Ethics Committee of the State of Brandenburg, Germany .

Note that full information on the approval of the study protocol must also be provided in the manuscript.
